# Transition Metal Activation Reframes SAMHD1 Regulation

**DOI:** 10.1021/acschembio.6c00438

**Published:** 2026-07-15

**Authors:** Logan A. Calderone, Anthony Gizzi, Soumika Pinninti, James T. Stivers, Maria-Eirini Pandelia

**Affiliations:** † Department of Biochemistry, 8244 Brandeis University, Waltham, Massachusetts 02453, United States; ‡ Department of Pharmacology and Molecular Sciences, Johns Hopkins University School of Medicine, Baltimore, Maryland 21205, United States

## Abstract

SAMHD1 is the lone human dNTP triphosphohydrolase and is linked to antiviral defense, nucleotide pool homeostasis, chemotherapy resistance, and the autoinflammatory Aicardi-Goutières syndrome. Although its substrate specificity and nucleotide-dependent oligomerization have been extensively studied, the identity and mechanistic roles of its metal cofactors remain poorly understood. Here, we integrate selective metal enrichment, spectroscopy, biochemical reconstitution, and enzyme kinetics to define the metal requirements underlying SAMHD1 activation and catalysis. We show that robust SAMHD1 activity is preferentially supported by transition metals and that the enzyme readily assembles multiple iron-containing dinuclear active sites in solution. Iron preferentially binds to one position of the bimetallic core and promotes recruitment of a second divalent metal required for catalysis. Although manganese can substitute for iron, it alters metal-binding equilibria and less efficiently supports dinuclear cofactor assembly, highlighting a specialized organizational role for iron. In contrast, the second site remains comparatively permissive and accommodates various divalent metal ions with distinct functional consequences. Mixed-metal active sites further retain catalytic activity across redox conditions that otherwise suppress activity in homodinuclear diiron configurations, suggesting that metal plasticity buffers SAMHD1 against oxidative inhibition. Transition metals additionally act as higher-affinity allosteric activators than Mg^2+^, revealing that metal identity contributes to both catalytic and regulatory layers of SAMHD1 function. Cumulatively, these findings redefine the metal requirements of SAMHD1 and establish a framework in which iron-dependent active site organization and mixed-metal flexibility cooperate to sustain dNTP hydrolysis under changing cellular environments and metal flux conditions.

## Graphical abstract

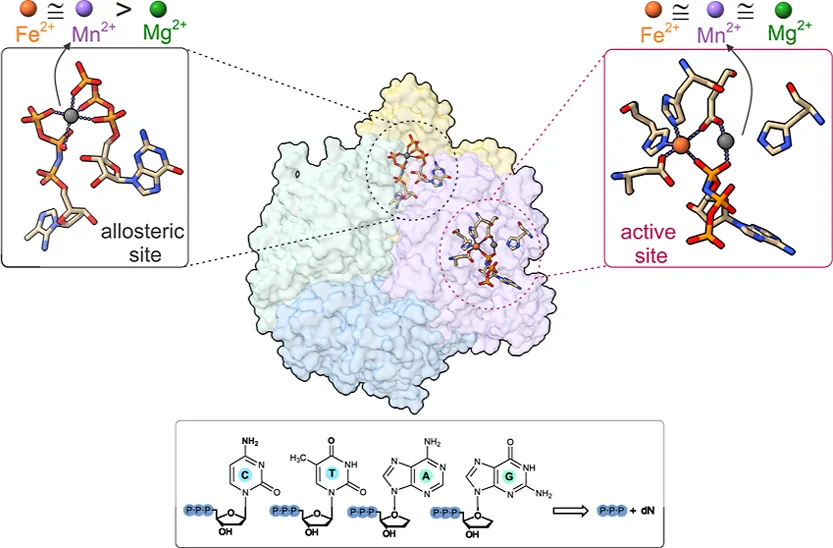

## Introduction

Metal ions are central determinants of enzymatic structure and catalysis, yet the identity and functional roles of metallocofactors frequently remain incompletely defined, particularly in iron-containing proteins.^1–4^ Although iron has classically been associated with oxidases and oxygenases, a growing number of nonredox enzymes, including phosphatases and phosphodiesterases, have also been shown to employ iron-dependent catalytic centers.^3,5–8^ This emerging role for iron is especially pronounced within the HD-domain superfamily, suggesting that transition-metal identity may contribute more broadly to hydrolytic catalysis and enzymatic regulation than previously appreciated.^9–14^

One HD-domain hydrolase in which iron has been identified as a constituent of the active site, but in which the identity and role of metals in activity remains poorly defined, is the sterile alpha motif and HD-domain containing protein 1 (SAMHD1).^15–20^ SAMHD1 is the only human deoxyribonucleotide triphosphate (dNTP) (metallo)­triphosphohydrolase and is intimately involved in restriction of viruses, including HIV, dNTP pool maintenance, resistance to chemotherapy, as well as the autoinflammatory Aicardi-Goutières syndrome.^16,17,21–31^ These diverse functions depend on metal-dependent oligomerization mediated by two allosteric sites, one (d)­GTP specific and the other dNTP dependent, as well as metal-dependent catalysis at the active site.^31–35^ While the structural determinants and functional consequences of oligomerization have been extensively studied, the identity, occupancy, and mechanistic role of the active and allosteric site metals remain incompletely defined. As a result, how metal coordination governs SAMHD1 assembly and enzymatic function remains poorly understood.

Here, we combine biochemical assays with metal-specific spectroscopic analyses of selectively metalated SAMHD1 forms to define the metal requirements underlying its dNTP phosphohydrolase activity. Using metal-depleted (apo) SAMHD1, we establish that catalytic activity strictly requires a transition metal ion. Reconstitution experiments further reveal that SAMHD1 readily incorporates iron, assembling either redox-active diiron or heterodinuclear active sites with other divalent metals. Within the bimetallic active site core one metal-binding site exhibits strong selectivity for iron, whereas the second site is more permissive, accommodating various divalent ions with functional consequences ranging from activation to inhibition. This metal tolerance extends to the allosteric sites, in which iron and manganese activate SAMHD1 with higher affinity than the canonical magnesium activator.^22,23,32–37^ Focusing on the active site, we show that the iron oxidation state does not greatly influence activity in heterodinuclear Fe-M^2+^ assemblies but controls catalysis in the homodinuclear Fe–Fe configurations. Moreover, iron uniquely promotes cooperative assembly of a dinuclear center, a property not recapitulated by manganese. Thus, iron acts as a critical determinant of both active-site architecture and function.

Collectively, our findings reveal that iron and transition-metal identity govern both active-site organization and allosteric regulation in SAMHD1. Moreover, mixed-metal active sites remain catalytically active under redox conditions that suppress activity in homodinuclear diiron configurations, suggesting that metal plasticity may buffer SAMHD1 against oxidative inhibition. In this context, iron may confer functional advantages in regulating dNTP catabolism, paralleling the role of mammalian ribonucleotide reductase, the iron-dependent enzyme responsible for de novo dNTP synthesis.^38–40^ Thus, iron may contribute to both the synthesis and degradation of deoxyribonucleotides in human cells, identifying metal identity as an underappreciated regulatory layer in nucleotide metabolism and antiviral defense.^25,26,37^ More broadly, defining how transition metals govern SAMHD1 assembly and catalysis provides a mechanistic framework for understanding its diverse cellular functions and may inform future strategies aimed at therapeutically modulating SAMHD1 activity in immune regulation, viral infection, and cancer.

## Results and Discussion

### SAMHD1 Catalysis Requires Transition Metal Ions

To determine the metal dependence of SAMHD1 activity, we generated a metal-depleted form of SAMHD1 (apo-SAMHD1) and assessed dGTP turnover in the presence of Mg^2+^, Fe^2+^, or Mn^2+^ (Figure 1A). In line with previous studies, no dNTP hydrolysis activity was detected in the absence of metal ions. Although Mg^2+^ is considered the canonical activator, its addition only resulted in a slow hydrolysis rate with minimal activity enhancement upon increasing its concentration.^22,23,32–37^ In contrast, Fe^2+^ and Mn^2+^ activated SAMHD1 with higher affinity and greater reaction velocities, demonstrating that these transition metals activate the enzyme more effectively (Figure 1A). Apo-SAMHD1 contained trace amounts of Fe^2+^ (Table S1), suggesting that the low activity observed upon Mg^2+^ supplementation likely arises from residual iron contamination.

**Figure 1:**
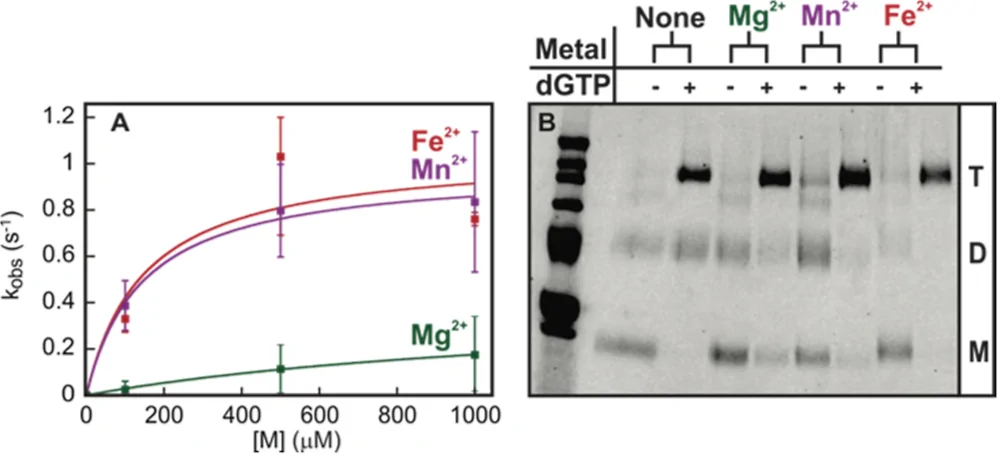
Metal dependence of oligomerization and activity in SAMHD1. (**A**). Dependence of apo-SAMHD1 activity on concentration of Fe^2+^ (red, activation constant *K*_act_ = 150 ± 168 μM), Mn^2+^ (purple, *K*_act_ = 146 ± 30 μM), or Mg^2+^ (green, *K*_act_ = 1350 ± 200 μM). The experiment was performed anaerobically under reducing conditions. (**B**). Glutaraldehyde cross-linking gel depicting oligomerization of SAMHD1 by Mg^2+^, Mn^2+^, or Fe^2+^, with and without dGTP. The T, D, and M labels indicate tetramer, dimer, and monomer, respectively. Error bars indicate the standard error of the mean (*n* = 2).

To assess the relationship between metal binding and oligomerization, we employed glutaraldehyde chemical cross-linking to confirm the SAMHD1 oligomeric state in the activity assays.^32,41^ In the absence of added metal ions, dGTP alone promoted partial tetramer formation, whereas addition of divalent metals further enhanced tetramerization (Figures 1B and S1). Because the dGTP stock was essentially metal free (Table S1), these observations indicate that nucleotide binding can drive a level of tetramerization independent of an allosteric metal, hitherto proposed to be Mg^2+^ coordinating the two nucleotides.^22,23,32–37^ However, full conversion to the tetrameric form required the presence of a metal ion, consistent with a concerted mechanism in which both nucleotide binding and metal coordination cooperate to stabilize the active oligomer.^22,33^ Both Fe^2+^ and Mn^2+^ markedly enhanced SAMHD1 catalytic activity and promoted formation of the tetramer. In contrast, Mg^2+^ supported stable tetramer assembly but resulted in only minimal catalytic turnover (Figure 1A,B). The data demonstrate that oligomerization alone is not sufficient for full enzymatic activation. Rather, robust catalysis requires iron or manganese. The limited activity observed in the presence of Mg^2+^ is consistent with catalysis arising from mixed-metal or incompletely occupied active sites, as suggested by recent crystal structures and elemental analyses (Table S1).^15,16,20,22,33^

These findings show that SAMHD1 catalysis is preferentially supported by transition metals, consistent with earlier structural studies that demonstrate the presence of a tightly bound Fe or Mn at the four coordinate HD-site.^22,23,32–37^ The data indicate that transition metals contribute directly to catalytic activation beyond their effects on oligomerization.^16,17,20^ In this context, SAMHD1 more closely resembles other metallophosphohydrolases in which transition metals, rather than Mg^2+^, drive catalysis.^3,9–13^

### SAMHD1 Assembles Diverse Iron-Containing Dinuclear Active Sites

Our experiments with apo-SAMHD1 combined with recent crystallographic data that revealed an Fe–Mg or Fe–Mn active site, suggest that SAMHD1 must be capable of assembling different dinuclear cofactors.^15,16,20,22,33^ To directly probe formation of such sites, we turned to EPR spectroscopy. Selective enrichment of SAMHD1 with iron was achieved by heterologous expression in minimal media supplemented with iron (M9-Fe) followed by isolation under O_2_-free conditions (Table S1). The samples were treated with sodium ascorbate to probe the accumulation of any mixed-valent paramagnetic states.^42–44^ Indeed, the EPR spectrum of the ascorbate-reduced M9-Fe SAMHD1 showed signals consistent with the formation of antiferromagnetically coupled (*S* = 1/2, *g*_av_ < 2) mixed-valence (Fe^3+^-Fe^2+^) diiron centers (Figure 2A).^42–46^ A minor signal at *g* = 4.3 was also detected, corresponding to a high-spin mononuclear Fe^3+^ species accounting for less than 5% of the total iron content (Figure 2B).^47^

**Figure 2:**
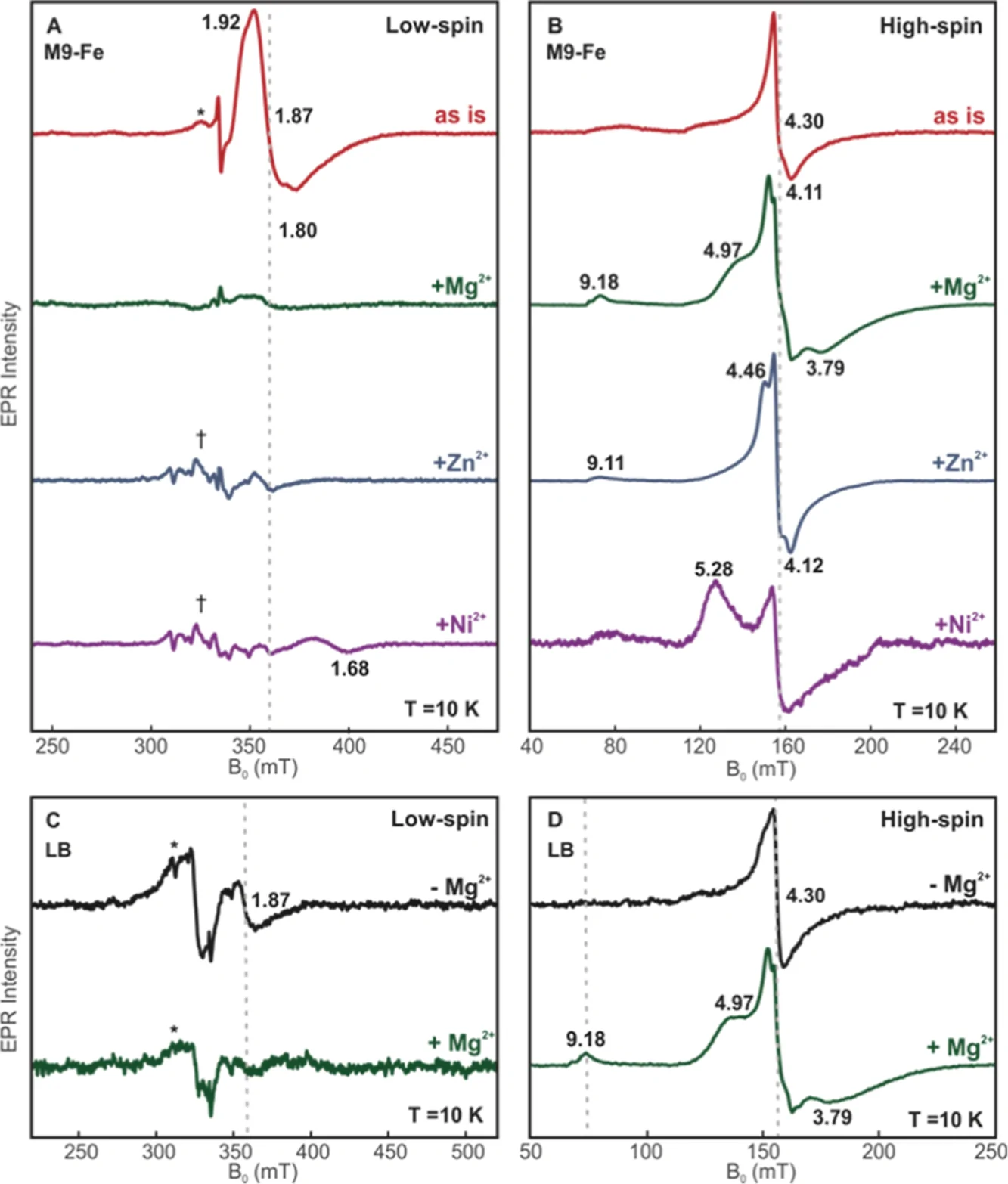
Monitoring assembly of a diiron cofactor and incorporation of various divalent metal ions at the active site of SAMHD1 by EPR. (**A**). EPR spectra of M9-Fe SAMHD1 as is (red) or with supplemental Mg^2+^ (green), Zn^2+^ (blue), or Ni^2+^ (purple) in the low-spin region. (**B**). EPR spectra of the same samples as in panel **A** in the high-spin region. (**C**). EPR spectra in the low-spin region of LB-SAMHD1 purified in either the absence (black) or presence (green) of Mg^2+^. (**D**). EPR spectra of the same samples in panel **C** in the high-spin region. Asterisks indicate signals from Cu^2+^ contamination. Daggers indicate signals from Mn^2+^ contamination. Experimental conditions: temperature = 10 K, microwave frequency = 9.38 GHz, microwave power = 2 mW, modulation amplitude = 1 mT.

Addition of divalent metal ions markedly altered the EPR spectra in both the low-spin and high-spin regions. Supplementation with Mg^2+^, Zn^2+^, or Ni^2+^ led to the loss of the *g*_av_ < 2 diiron signal and the appearance of new high-spin resonances near the mononuclear Fe^3+^ region (Figure 2A,B). Notably, these resonances exhibited pronounced rhombicity distinct from that of the isolated mononuclear Fe^3+^ species (Figure S2A-B). The spectral changes are consistent with displacement of diiron centers and formation of mixed-metal, iron-containing dinuclear sites.^48–50^ Specifically, addition of Mg^2+^ produced signals at *g* = 9.18, 4.97, and 3.79 arising from excited and ground state Kramers’ doublets, which are consistent with changes in the rhombicity of the high-spin Fe^3+^ due to its complexation with a divalent metal (Figures 2B and S2B). Addition of Zn^2+^ resulted in the appearance of a high-spin feature at *g* ∼9.11 as well as additional resonances in the region of the mononuclear Fe^3+^ signal, while addition of Ni^2+^ resulted in a rhombic signal with apparent *g* values of 5.28 and 1.68 (Figure 2B). These observations demonstrate that SAMHD1 readily remodels its dinuclear active site in response to metal availability.

To evaluate whether similar metal exchange occurs under conventional purification conditions, we analyzed SAMHD1 purified from LB media with or without Mg^2+^ present in the purification buffers. Omission of Mg^2+^, preserved a weak mixed-valent *g*_av_ < 2 signal, consistent with formation of diiron centers (Figure 2C). In contrast, inclusion of Mg^2+^ in the purification buffers, as employed in prior protocols,^16,22,23,51^ abolished this mixed-valence Fe^3+^-Fe^2+^ signal and produced spectra resembling those of Mg^2+^-treated M9-Fe SAMHD1 (Figure 2D). These data provide direct evidence that the metal configurations previously inferred from crystal structures are populated in solution and are strongly influenced by purification conditions and metal availability.^16,17,20^

Our spectroscopic and metal reconstitution experiments are consistent with a model in which iron preferentially occupies one position within the dinuclear center and promotes recruitment of the second metal ion required for catalysis. Collectively, these findings establish that SAMHD1 assembles multiple iron-containing dinuclear active sites in solution and that iron-containing cofactors are readily remodeled by competing divalent metals.

### Manganese-Dependent Cofactor Assembly and Catalytic Activation

Having established that iron directs the assembly of multiple dinuclear cofactors in SAMHD1, we next examined how perturbation of the metal-binding environment influences cofactor integrity. Guided by crystallographic models identifying His233 as a ligand of the more permissive metal site, we used EPR spectroscopy to assess the impact of removing this residue.^16,17,20^ Substitution of H233 with alanine abolished formation of the dinuclear cofactor, confirming its essential role in metal coordination and cofactor assembly (Figure 3A,B).

**Figure 3:**
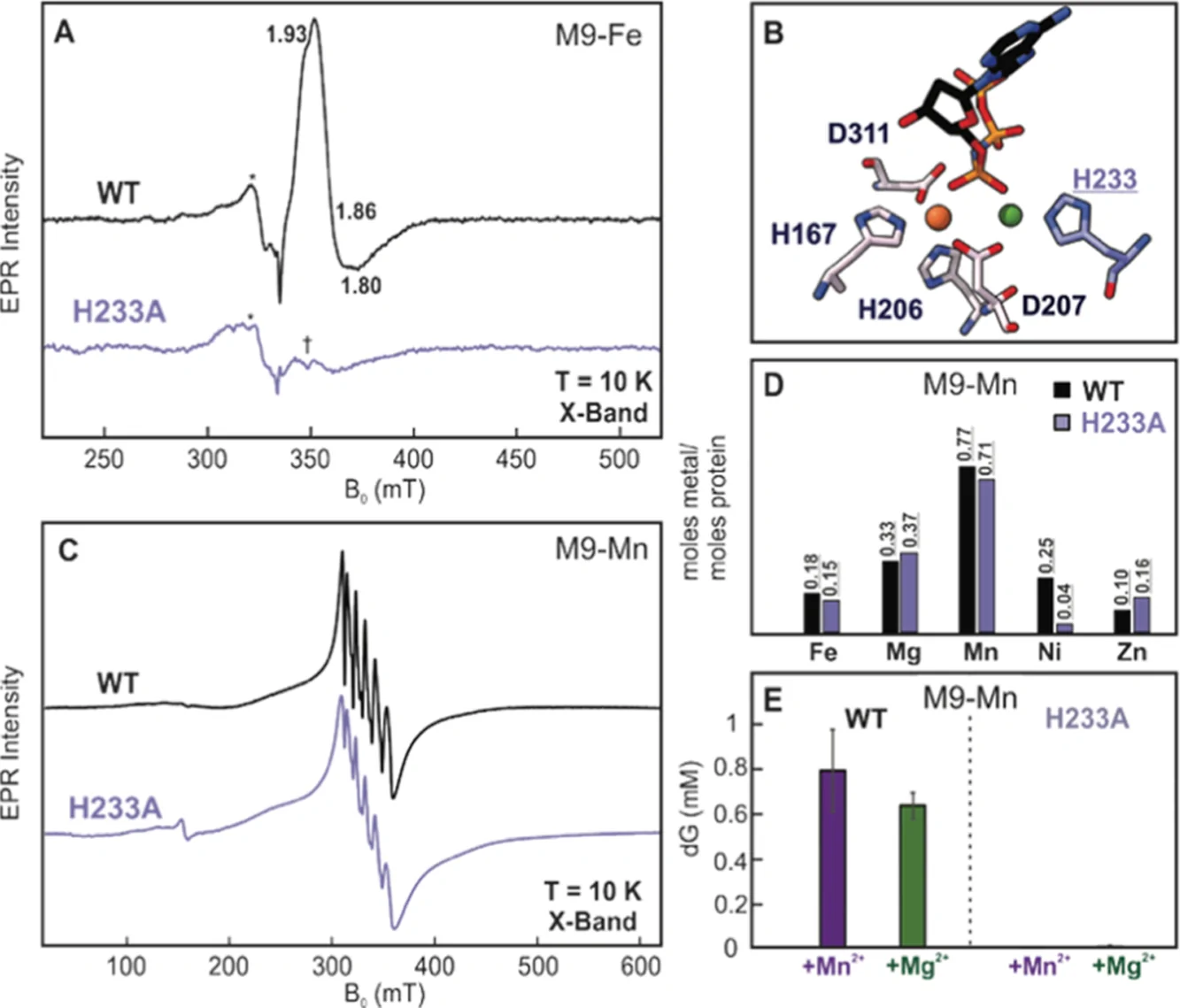
Spectroscopic characterization of wild-type and H233A SAMHD1 under different metalation enrichment, metal content and catalytic activity of Mn-enriched SAMHD1. (**A**). EPR spectra of wild-type and H233A SAMHD1 enriched with iron (M9-Fe). Asterisks indicate signals from Cu^2+^ contamination. Daggers indicate signals from Mn^2+^ contamination. (**B**). Active site structure of the *Hs* SAMHD1 with 2′-deoxy-5′-*O*-[(R)-hydroxy­([(R)-hydroxy­(phosphonooxy)­phosphoryl]­amino)­phosphoryl] adenosine bound (PDB ID: 6TX0). The Fe ion is shown as an orange sphere and the Mg ion as a green sphere. Only the primary coordinating ligands are shown with the H233 ligand to the Mg ion (site 2) highlighted in purple. (**C**). EPR spectra of wild-type and H233A SAMHD1 enriched with manganese (M9-Mn). (**D**). ICP-AES data comparing the molar ratios of metals that copurify with wild-type (black bars) and H233A M9-Mn SAMHD1 (purple bars). (**E**). HPLC end point activity assays comparing the activity of wild-type and H233A M9-Mn SAMHD1 with supplemental Mn^2+^ (purple bars) or Mg^2+^ (green bars). Experimental conditions: temperature = 10 K, microwave frequency = 9.38 GHz, microwave power = 2 mW, modulation amplitude = 1 mT. Activity assays were carried out with [*Hs* SAMHD1] = 0.5 μM, [dGTP] = 1 mM, and [Mn] = 2 mM or [Mg] = 2.5 mM for 10 min. Error bars indicate the standard error of the mean (*n* = 4).

To examine the effect of manganese on cofactor composition, wild-type and H233A SAMHD1 was selectively enriched with Mn^2+^ via heterologous expression in minimal media (M9-Mn, Table S1). EPR analysis revealed spectra dominated by a well-resolved six-line hyperfine pattern characteristic of mononuclear Mn^2+^ centers, rather than the broadened multiline features expected for dimanganese sites (Figure 3C).^52,53^ This spectral signature was reproducible across multiple preparations, indicating that formation of a dimanganese cofactor is disfavored under these conditions and suggesting that Mn^2+^ occupancy at one site influences metal binding at the second one. The Mn^2+^-enriched H233A variant exhibited EPR spectra comparable to those of the wild-type enzyme, and ICP-AES analysis confirmed similar overall manganese content in both proteins (Figure 3D, Table S1). Together, these data support a model in which manganese preferentially occupies the 4-coordinate HD site, whereas the H233-associated site displays reduced affinity for Mn^2+^ (Figure 3B).^16,17,20^

Consistent with our previous observation that apo-SAMHD1 supplemented with Mn^2+^ is catalytically active (Figure 1A), addition of Mn^2+^ or Mg^2+^ to wild-type M9-Mn SAMHD1 restores activity to rates comparable to those reported previously (0.5–5.0 s^–1^) (Figure 3E, Table S2).^16,32,54^ Although Mn^2+^-enriched SAMHD1 purified under these conditions predominantly harbors mononuclear Mn^2+^ centers, inclusion of excess Mn^2+^ (or Mg^2+^) during the activity assays promotes formation of catalytically competent dimetal sites. In contrast, the H233A M9-Mn variant remained inactive with Mn^2+^ (Figure 3E, Table S2), indicating that catalytic activity depends on assembly of a dinuclear cofactor and cannot be supported when coordination at the second metal-binding site is disrupted. Therefore, Mn^2+^ supports activity when a second metal can bind, but it preferentially forms mononuclear species and does not organize the dinuclear center as effectively as iron.

Thus, iron and manganese make distinct contributions to SAMHD1 metalation. Whereas iron readily promotes assembly of stable dinuclear centers, manganese preferentially accumulates as mononuclear species and requires additional metal supplementation to support robust catalysis. In this context SAMHD1 parallels other HD-domain phosphohydrolases and purple acid phosphatases, in which a tightly bound iron ion stabilizes the active-site architecture and promotes binding of a second catalytic metal.^9,13^

### Catalytic and Allosteric Metals Independently Tune SAMHD1 Activity

To evaluate the impact of active-site metal identity on SAMHD1-catalyzed dNTP hydrolysis, we systematically varied the second metal in the dinuclear active center while keeping the allosteric effector constant (Figure 4). We examined Fe^2+^-M^2+^ active-site configurations, where M was Mg^2+^, Mn^2+^, or Fe^2+^, using Mg^2+^ as the allosteric effector. All samples were prereduced with sodium dithionite to ensure that iron remained in the ferrous state for consistency. EPR spectroscopy confirmed that the excess Mg^2+^ added as effector did not exchange into the active site or alter its metal composition over the course of the assay (Figures S3 and S4). Because metal incorporation differed among preparations, catalytic rates were normalized to the iron content of each sample. Under these conditions, the Fe^2+^-Mg^2+^ form hydrolyzed dGTP with an activation constant (*K*_act_) of 257 ± 80 μM and an apparent rate constant of 1.8 ± 0.2 s^–1^, comparable to previously reported measurements (Figure 4A, Table S3).^16,32,54^ The homodinuclear Fe^2+^-Fe^2+^ cofactor supported indistinguishable catalytic activity and a similar activation constant, indicating that this configuration is fully competent for catalysis. In contrast, the Fe^2+^-Mn^2+^ form displayed a comparable *K*_act_ but slower catalytic turnover, suggesting a lower turnover efficiency under these conditions (Figure 4A, Table S3). It should be noted that this preparation contained higher levels of Ni^2+^ than the others, which may partially suppress the observed rates (Table S1). Together, these results show that the chemical nature of the divalent metal at the second site does not substantially affect activation, and maximal catalytic rates are achieved with Fe^2+^-Fe^2+^ and Fe^2+^-Mg^2+^ active-site configurations.

**Figure 4:**
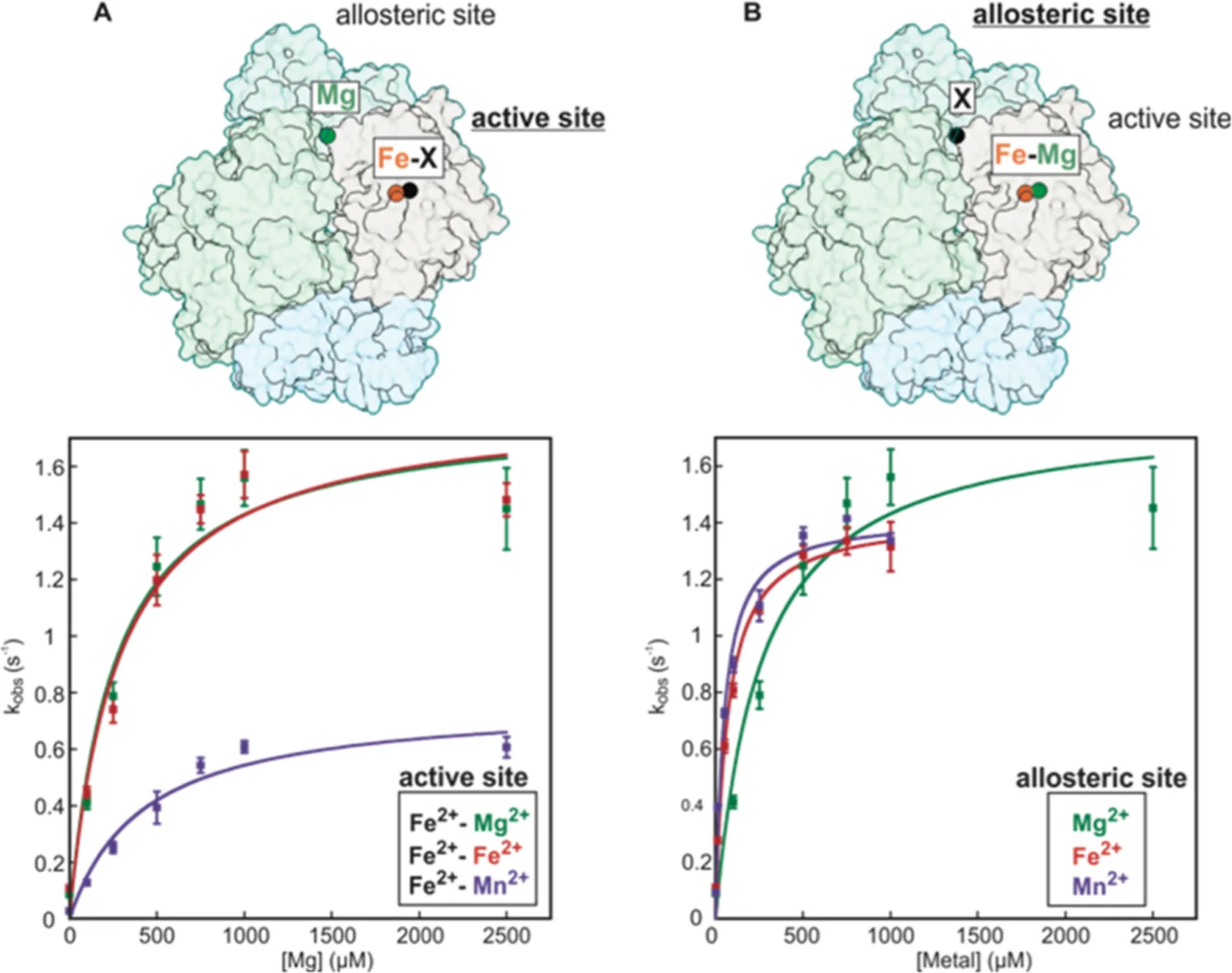
SAMHD1 activity assays highlight metal plasticity at the active and allosteric sites. (**A**). HPLC activity assay of SAMHD1 enriched with Fe^2+^-Mg^2+^ (M9-FeMg, green, *k*_obs_ = 1.80 ± 0.2 s^–1^, *K*_act_ = 257 ± 80 μM), Fe^2+^-Fe^2+^ (M9-Fe, red, *k*_obs_ = 1.82 ± 0.2 s^–1^, *K*_act_ = 277 ± 80 μM), or Fe^2+^-Mn^2+^ (M9-Fe/Mn, purple, *k*_obs_ = 0.77 ± 0.08 s^–1^, *K*_act_ = 417 ± 120 μM) using magnesium as an allosteric activator. (**B**). HPLC activity assay of SAMHD1 enriched with Fe^2+^-Mg^2+^ (M9-Fe/Mg) using Mg^2+^ (green, *k*_obs_ = 1.80 ± 0.2 s^–1^, *K*_act_ = 257 ± 80 μM), Fe^2+^ (red, *k*_obs_ = 1.42 ± 0.05 s^–1^, *K*_act_ = 68 ± 10 μM), or Mn^2+^ (purple, *k*_obs_ = 1.42 ± 0.07 s^–1^, *K*_act_ = 47 ± 10 μM) as allosteric activators. Error bars indicate the standard error of the mean (*n* = 3).

These observations demonstrate that the second metal-binding site tolerates multiple divalent metals while preserving catalytic activity. The data further suggest that iron primarily functions as an organizing element within the dinuclear center, whereas the second site modulates catalytic efficiency without fundamentally altering activity. Such metal plasticity may be a broader feature of HD-domain enzymes, enabling function across fluctuating metal availability.^13,14,55^ Consistent with this model fungal SAMHD1 orthologs and the bacterial phosphohydrolase YqeK (both belonging to the HD-domain superfamily) similarly support catalytically active homometallic and mixed-metal dinuclear active sites, in which iron serves as a central architectural cofactor element.^13,55^

We next examined the contribution of the allosteric metal effector to catalysis. Using the Fe^2+^-Mg^2+^ active-site configuration, dGTP hydrolysis was measured while varying the type of allosteric activator between Mg^2+^, Fe^2+^, and Mn^2+^ (Figure 4B). Activation with Fe^2+^ supported a comparable maximal rate (*k*_cat_ = 1.42 ± 0.05 s^–1^) but with an approximately 4-fold lower *K*_act_ (68 ± 10 μM) compared to Mg^2+^ (*K*_act_ = 257 ± 80 μM), indicating tighter allosteric activation. Furthermore, Mn^2+^ produced nearly identical kinetic parameters to Fe^2+^ (Table S3), demonstrating that transition metals can function as higher-affinity allosteric activators than Mg^2+^ and may therefore be physiologically relevant under cellular conditions. Together, these findings demonstrate that SAMHD1 tolerates multiple active-site metal configurations while remaining highly responsive to transition metals at the allosteric site. Iron therefore contributes both to active-site organization and to efficient allosteric activation, suggesting that intracellular metal availability may influence SAMHD1 activity under physiological conditions.

Although intracellular Mg^2+^ concentrations generally exceed those of transition metal ions, occupancy at the allosteric site and the more labile position of the dinuclear active center are determined by both free metal concentration in cells and binding affinity. The substantially lower *K*_act_ values observed for Fe^2+^ and Mn^2+^ demonstrate that SAMHD1 is more efficiently activated by transition metal ions than by Mg^2+^. Moreover the comparable catalytic activities observed for homo- and heterobimetallic Fe sites, suggest that multiple metalation states of SAMHD1 may be physiologically relevant. Therefore, despite the higher cellular abundance of Mg^2+^, transition metal ions can compete for SAMHD1 activation, particularly in macrophages and other iron-responsive immune cells in which intracellular iron availability and redox conditions fluctuate during infection and immune activation.^56–63^

### Mixed-Metal Active Sites Buffer SAMHD1 against Redox-Dependent Inhibition

Because iron-containing cofactors can populate multiple oxidation states, we next examined whether iron redox chemistry influences SAMHD1 catalysis. This question is particularly relevant because SAMHD1 is expressed in macrophages,^26,56,58,64–66^ a comparatively oxidizing cellular environment, and oxidation to the ferric state is inhibitory in hydrolases.^3^ To define the accessible redox states of the SAMHD1 diiron cofactor, we employed ^57^Fe Mössbauer spectroscopy (Figure 5A, Table S4). The Mössbauer spectra revealed that anaerobically purified SAMHD1 contained a mixture of oxidation states, consisting predominantly of the diferric (Fe^3+^-Fe^3+^, ∼50%) and mixed-valent (Fe^3+^-Fe^2+^, ∼30%) species (Table S4). The remaining spectral intensity could not be completely accounted for due the broad background. Upon reduction with ascorbate, the mixed-valent Fe^3+^-Fe^2+^ state became the dominant species (∼56%), consistent with the EPR experiments (Figure 2). In this sample, a minor fraction of the diferric state (∼10%) persisted, while the fully reduced diferrous (Fe^2+^-Fe^2+^) form accounted for ∼30% of the iron population (Table S4). Treatment with sodium dithionite resulted in quantitative conversion to the diferrous state. Thus, the SAMHD1 diiron cofactor populates diferric, mixed-valent, and diferrous states that can be interconverted by mild redox treatments (Figure 5A, Table S4). This behavior closely parallels that reported for other HD-domain diiron enzymes and provides a framework to assess how the iron oxidation state influences catalytic function.^11–13,42,43,55^

**Figure 5:**
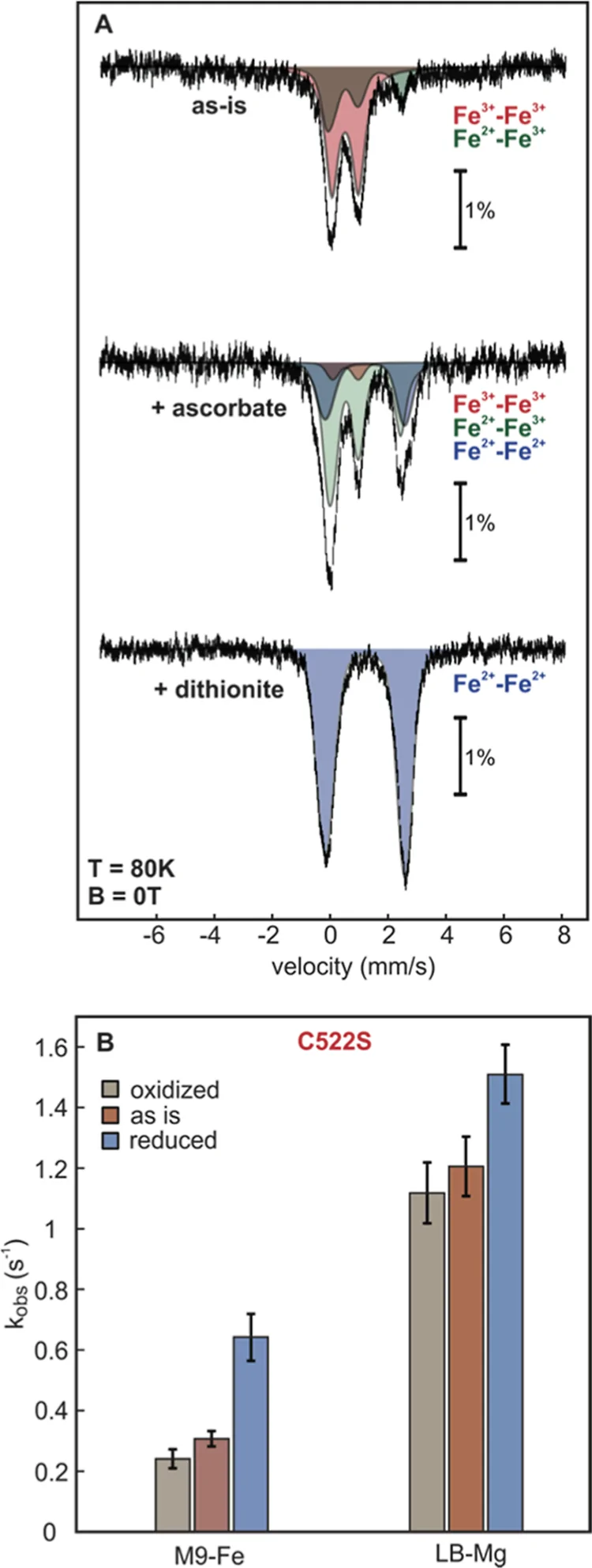
Distribution of redox states in Fe-enriched SAMHD1 by Mössbauer spectroscopy and redox-dependence of hydrolysis. (**A**). Mössbauer spectra of M9-Fe SAMHD1 under various redox conditions showing individual spectra of the Fe^3+^-Fe^3+^ species (red), the Fe^3+^-Fe^2+^ species (green), and the Fe^2+^-Fe^2+^ species (blue). Overlapping spectra are shown as the convolution of the underlying colors. Raw data is displayed as black vertical bars. (**B**). HPLC activity assay data of M9-Fe C522S SAMHD1 and LB-Mg C522S SAMHD1 under oxidizing (gray), as is (brown), or reducing conditions (blue). Error bars indicate the standard error of the mean (M9-Fe oxidized, as is, *n* = 5), (M9-Fe reduced, *n* = 6). (LB-Mg oxidized, as is, *n* = 7), (LB-Mg reduced, *n* = 8).

To explore the effects of the Fe redox state on the overall catalytic activity of SAMHD1 we employed M9-Fe C522S SAMHD1 (Figure 5B, Table S1). The C522S variant bypasses the known inhibition of the wild-type enzyme under oxidative conditions due to disulfide formation involving C522, while retaining wild-type activity.^67–69^ The as isolated protein hydrolyzed dGTP with an apparent rate of 0.31 ± 0.03 s^–1^, while treatment with hydrogen peroxide resulted in a slight decrease of the hydrolysis rate (Figure 5B, Table S5). This persistent background activity even in an oxidizing environment likely stems from residual Mg^2+^ contamination that could not be fully eliminated (Table S1). In contrast, reduction with sodium dithionite, which produces homogeneous enrichment in diferrous centers, resulted in an approximately 2-fold increase in the hydrolysis rate (Table S5).

We next asked whether a similar redox dependence is observed for heterodinuclear active sites. To this end, the Fe–Mg-enriched C522S SAMHD1 variant was examined under the same redox conditions. Oxidation with hydrogen peroxide, which maximizes the Fe^3+^-Mg^2+^ configuration, supported dGTP hydrolysis with an apparent rate of 1.12 ± 0.1 s^–1^, only slightly lower than that of the as-isolated enzyme (Table S5). Further, reduction with sodium dithionite led only to a modest increase in activity. Thus, in contrast to the diiron form, catalytic activity of the Fe–Mg^2+^ center is not largely affected by the oxidation state.

Together, these data reveal that the susceptibility of SAMHD1 to redox-dependent inhibition shows a detectable dependence on the identity of the second metal ion. Diiron active sites exhibit pronounced redox sensitivity, with maximal activity in the diferrous state, whereas mixed-metal Fe–Mg^2+^ centers retain robust activity even under oxidizing conditions. These observations also imply that the mixed-valent Fe^3+^-Fe^2+^ state remains catalytically competent, consistent with our EPR experiments showing substrate binding to both the Fe^3+^-Fe^2+^ and Fe^3+^-Mg^2+^ forms (Figures S4 and S5).

The catalytic consequences of iron oxidation state in SAMHD1 thus depend on the identity of the second metal ion. Diiron centers display pronounced redox sensitivity, with maximal activity observed in the diferrous state, whereas incorporation of a redox-inert metal such as magnesium buffers the enzyme against oxidative inactivation. These findings provide direct experimental evidence that redox state influences SAMHD1 catalysis and suggest that mixed-metal active sites preserve enzymatic function under oxidizing cellular conditions.

The metal dependence uncovered here also has broader implications for nucleotide metabolism. Mammalian ribonucleotide reductase, which catalyzes de novo dNTP synthesis, is itself a diiron enzyme.^38,40^ Our finding that SAMHD1 also relies on iron for efficient dNTP hydrolysis raises the possibility that iron availability could regulate both the synthesis and degradation of deoxyribonucleotides. Although ribonucleotide reductases across biology use diverse metallocofactors,^39,40^ the mammalian context suggests an intriguing convergence in which iron contributes to multiple points of dNTP control required for genome stability and antiviral defense.

In conclusion, our results redefine the metal requirements of SAMHD1 and reveal a metalloregulatory framework for dNTP hydrolysis. Iron emerges as a central determinant of active site assembly, while promiscuity at the second metal-binding site preserves enzymatic function across diverse cellular metal and redox conditions. Beyond its structural role, iron also acts as a high-affinity allosteric activator, linking metal availability to regulation of SAMHD1 activity. More broadly, these results position SAMHD1 within a growing class of transition-metal-regulated HD-domain hydrolases and suggest that transition metals in general and iron in particular represent an underappreciated regulatory layer in nucleotide metabolism and antiviral defense.

## Methods

### Materials

All chemicals were of high-purity grade and obtained from Fisher Scientific unless otherwise specified.

#### Human SAMHD1 Expression

For protein expression, *Escherichia coli* T7 express cells (New England Biolabs) were transformed with a previously constructed pET-19b plasmid containing the wild-type and variant His_6_-tagged SAMHD1 (Δ112) from *Homo sapiens* and an ampicillin resistance marker.^18^ The cells were grown in either Luria–Bertani (LB) or minimal (M9) media to direct specific metal incorporation. In the case of M9 media the cultures contained 0.5% (w/v) glucose, 90 μM thiamine hydrochloride, 0.1 mM CaCl_2_, 2 mM MgSO_4_, and different transition metal ion salts: 125 μM (NH_4_)_2_Fe­(SO_4_)_2_ or 100 μM MnCl_2_, for M9-Fe and M9-Mn, respectively. The cells were incubated at 37 °C and 220 rpm until an OD_600_ of 0.6–1.0 was reached. The cultures were cold shocked for 1 h at 4 °C and protein expression was induced by addition of 0.5 mM Isopropyl β-*d*-1-thiogalactopyranoside (IPTG), and 125 μM (NH_4_)_2_Fe­(SO_4_)_2_ or 100 μM MnCl_2_, respectively. After shaking (220 rpm) for 16–20 h at 18 °C, the cells were harvested by centrifugation at 1,600*g* and 4 °C for 20 min, flash frozen in liquid nitrogen, and stored at −80 °C prior to further use.

### Human SAMHD1 Purification

In an O_2_-free glovebox (Coylab), cell pellets were resuspended in lysis buffer (50 mM 4-(2-Hydroxyethyl)­piperazine-1-ethanesulfonic acid (HEPES), 300 mM potassium chloride (KCl), 10 mM imidazole, 0.5 mM tris­(2-carboxyethyl) phosphine (TCEP), 10% glycerol, pH 7.5) and lysed using a Qsonica sonicator with the addition of 10 mg/L lysozyme, and 0.45 μg/L phenylmethylsulfonyl fluoride (PMSF) prior to cell disruption. Lysed cells were centrifuged at 40,000*g* for 30 min. The clarified lysate was loaded onto a Ni^2+^-NTA immobilized affinity chromatography column (∼10 mL resin per 100 mL lysate) equilibrated with the lysis buffer. The column was first washed with lysis buffer and then with wash buffer (50 mM HEPES, 300 mM KCl, 30 mM imidazole, 0.5 mM TCEP, 10% glycerol, pH 7.5). The protein was eluted with elution buffer (50 mM HEPES, 300 mM KCl, 250 mM imidazole, 0.5 mM TCEP, 10% glycerol, pH 7.5). When SAMHD1 was purified in the presence of magnesium (LB-Mg, M9-Fe/Mg) or manganese (M9-Fe/Mn), 4 mM MgCl_2_ or MnCl_2_ respectively were included in the lysis, wash, and elution buffers. The elution was concentrated at 3900 rpm using a 50 kDa MWCO Amicon Centrifugal Filter Unit (Millipore Sigma, MA). The concentrated protein was loaded onto a HiLoad 16/600 Superdex 200 pg (Cytiva, MA) size exclusion column, which was equilibrated with storage buffer (50 mM HEPES, 300 mM KCl, 0.5 mM TCEP, 10% glycerol, pH 7.5).^18^ Fractions containing the pure protein were pooled and further concentrated. Protein purity was estimated by SDS-PAGE with Coomassie staining, and protein concentration was determined using a molar absorption coefficient of 63,260 M^–1^ cm^–1^ at 280 nm (https://web.expasy.org/protparam/) via an Ultraviolet–visible (UV–vis) spectrophotometer (Agilent, CA). The protein was flash-frozen in liquid nitrogen and stored at −80 °C in airtight cryovials prior to further use.

### SAMHD1 Chelation

M9-Fe SAMHD1 was reduced with 10 mM sodium dithionite (NaDT) for 10 min in an anaerobic glovebox. Dipicolinic acid (DPA) was then added to a concentration of 1.25 mM, and the protein was left to react at 4 °C overnight. Removal of the NaDT and DPA was achieved under O_2_-free conditions using a NAP-5 desalting column (Cytiva). To ensure optimal chelation, this process was repeated once.

### SAMHD1 Activity Assays

All activity assays were carried out under O_2_-free conditions in an anaerobic glovebox and in the presence of NaDT, unless otherwise specified. SAMHD1 containing storage buffer was preincubated with 10 mM NaDT for 10 min and aliquoted into a 96-well plate. The reactions were initiated by addition of buffer containing the allosteric metal as well as dGTP, the allosteric nucleotide and substrate, to bring the final concentrations in the reaction to 0.5 μM SAMHD1, 0.5 mM NaDT, and 1 mM dGTP, as well as a variable concentration of the allosteric metal (0–5 mM). The reaction was quenched by 1:1 dilution with the quench solution containing a 270 μM caffeine internal standard (ε_260_ = 12,399 M^–1^ cm^–1^) and 100 mM potassium hydroxide at 2.5, 5, and 10 min, respectively. For the redox-dependent assays, as is SAMHD1 was not preincubated with 10 mM NaDT and oxidized SAMHD1 was preincubated with 7.5 mM hydrogen peroxide, respectively.

### HPLC Analysis

SAMHD1 activity assay samples were analyzed on an Agilent 1260 Infinity II high-performance liquid chromatography system equipped with a 1260 Infinity Photodiode Array Detector WR by monitoring the absorbance at 260 nm.^55^ The samples were injected on a 150 × 4.6 mm Phenomenex Synergi 4u Fusion-RP 80 Å pore size reverse phase column and run at 60 °C using aqueous (20 mM ammonium acetate pH 4.5, Solvent A) and organic (methanol, Solvent B) mobile phases in a 19 min program consisting of an 11 min gradient from 100% A to 21.5% A and 78.5% B, which ran for a further one min followed by a three min gradient from 21.5% A and 78.5% B to 100% B and a jump to 100% A for the final four min.^51^ The amount of product (dG) formation was quantified by comparing the integrated absorbances of the dG (ε_260_ = 11,500 M^–1^ cm^–1^) peak to the caffeine (ε_260_ = 12,399 M^–1^ cm^–1^) internal standard peak, and normalizing for their different extinction coefficients at 260 nm.

### Glutaraldehyde Cross-Linking

The glutaraldehyde cross-linking was carried out following previously published procedures with some modifications.^41^ First, 1 μM SAMHD1 was incubated in the cross-linking buffer (50 mM HEPES, 50 mM KCl, pH 7.5) alongside 200 μM dGTP or 1 mM metal, if necessary, for 20 min. The iron condition was performed in an anaerobic glovebox and reduced with 2 mM NaDT to ensure that all the iron was in the ferrous state. Next, 20 μL of 50 mM glutaraldehyde, in water, was added to each sample, and they were incubated at RT for 15 min. The cross-linking reaction was quenched by addition of 8 μL of 1 M *tris*(hydroxymethyl) aminomethane hydrochloride (Tris–HCl) pH 7.5. Finally, 16 μL of 4x Laemmli loading dye was added to each sample, and they were run on Invitrogen NuPAGE 4*-*12%, Bis-Tris, 1.5 mm, 10-well precast gels. The gels were imaged on a ChemiDoc Imager (Bio-Rad).

### Electron Paramagnetic Resonance

All samples were prepared in storage buffer (50 mM HEPES, 300 mM KCl, 10% glycerol, 0.5 mM TCEP, pH 7.5) under O_2_-free conditions in an anaerobic glovebox. Reduction with sodium ascorbate was performed with 2 molar eq (with respect to protein concentration) for 10 min at RT prior to freezing in liquid nitrogen. EPR spectra were acquired at 10 K on a Bruker E500 Elexsys continuous wave (CW) X-Band spectrometer (operating at approximately 9.36 GHz) equipped with a rectangular resonator (TE102) and a continuous-flow cryostat (Oxford 910) with a temperature controller (Oxford ITC 503).

### Electron Paramagnetic Resonance Metal Replacement Studies

To monitor metal replacement at the active site of SAMHD1, M9-Fe SAMHD1 was incubated overnight with excess metal salts (100 mM MgCl_2_ 1 mM ZnSO_4_, or 10 mM NiSO_4_). These concentrations were chosen to promote maximal displacement of the second Fe ion while maintaining protein stability. Samples were subsequently frozen in liquid nitrogen and analyzed as described above.

### Inductively Coupled Plasma Atomic Emission Spectrometry

Metal analysis of the protein samples was determined by inductively coupled atomic emission spectrometry (ICP-AES) at the Energy and Environmental Sustainability Laboratories (EESL) at the Pennsylvania State University (PA). Samples were prepared by precipitation in 3.5% metal-free nitric acid.

### Ferrozine Assays

Iron stoichiometry was also determined using the colorimetric ferrozine assay. The protein of interest was diluted to 15 μM and precipitated by addition of trichloroacetic acid (Ricca, Tx) to a final concentration of 8% (w/v). After incubation for 20 min, samples were spun at 13,000*g* for 2 min and the supernatant was removed and diluted to a final volume of 500 μL with water. To this sample, 2.3 mM of ascorbic acid, 300 μM of ferrozine, and 18% (v/v) of a saturated ammonium acetate solution were added. The sample was then mixed and the Fe^2+^-ferrozine complex was monitored at 562 nm (ε_562_ = 26,385 M^–1^ cm^–1^).

### ^57^Fe Mössbauer Spectroscopy

Mössbauer samples were prepared from M9-Fe SAMHD1 in storage buffer (50 mM HEPES, 300 mM KCl, 10% glycerol, 0.5 mM TCEP, pH 7.5) under O_2_-free conditions. Samples were reacted with 2 mol eq of sodium ascorbate or 10 mM NaDT for 15 min as indicated. Mössbauer spectra were recorded on a WEB Research (Edina, MN) instrument. The spectrometer used to acquire the weak-field spectra is equipped with a Janis SVT-400 variable-temperature cryostat. The external magnetic field was applied parallel to the γ beam. All isomer shifts are quoted relative to the centroid of the spectrum of α-iron metal at RT. Mössbauer spectra were fit using the WMOSS4Mössbauer Spectral Analysis Software (www.wmoss.org).

## Supplementary Material



## Notes

Additional experimental details, materials, and methods, Figure S1: Glutaraldehyde cross-linking experiments, Figure S1: EPR Rhomograms, Figures S2–S5: EPR spectra of SAMHD1 under different conditions, Table S1: ICP-AES elemental analyses values, Table S2–S4: Sprectroscopic and kinetic parameters of different SAMHD1 forms (PDF)
